# Supplementary material for: Developmental signals control chromosome segregation fidelity during pluripotency and neurogenesis by modulating replicative stress
Source: Nat Commun. 2024 Aug 28;15:7404. doi: 10.1038/s41467-024-51821-9 (PMC11350214; doi:10.1038/s41467-024-51821-9)
Supplement: Supplementary file 1 — Supplementary Information [file 41467_2024_51821_MOESM1_ESM.pdf]

Supplementary information

Developmental signals control chromosome segregation fidelity during pluripotency and neurogenesis by modulating replicative stress

Anchel de Jaime-Soguero[1,15], Janina Hattemer[1,15], Anja Bufe[1], Alexander Haas[2], Jeroen van den Berg[3,4,5,6], Vincent van Batenburg[3,4,5,6], Biswajit Das[7], Barbara di Marco[8], Stefania Androulaki[1], Nicolas Böhly[2], Jonathan J.M. Landry[9], Brigitte Schoell[10], Viviane S. Rosa[11], Laura Villacorta[9], Yagmur Baskan[1], Marleen Trapp[12], Vladimir Benes[9], Andrei Chabes[7], Marta Shahbazi[11], Anna Jauch[10], Ulrike Engel[13], Annarita Patrizi[12], Rocio Sotillo[14], Alexander van Oudenaarden[3,4,5,6], Josephine Bageritz[1], Julieta Alfonso[8], Holger Bastians[2,16] and Sergio P. Acebrón[1,16,*]

[1] Centre for Organismal Studies (COS), Heidelberg University, D-69120 Heidelberg, Germany

[2] University Medical Center Göttingen (UMG), Department of Molecular Oncology, Section for Cellular Oncology, D-37077 Göttingen, Germany

[3] Oncode Institute, 3521 AL, Utrecht, The Netherlands

[4] Hubrecht Institute, 3508 AD, Utrecht, The Netherlands

[5] KNAW (Royal Netherlands Academy of Arts and Sciences), Utrecht, The Netherlands

[6] University Medical Center Utrecht, Utrecht, The Netherlands

[7] Department of Medical Biochemistry and Biophysics, Umeå University, 901 87 Umeå, Sweden

[8] Department of Clinical Neurobiology, University Hospital Heidelberg and German Cancer Research Center (DKFZ), D-69120 Heidelberg, Germany

[9] Genomics Core Facility, European Molecular Biology Laboratory (EMBL), Heidelberg, Germany

[10] Institute of Human Genetics, Heidelberg University, D-69120 Heidelberg, Germany

[11] MRC Laboratory of Molecular Biology, CB2 0QH Cambridge, UK

[12] Schaller Research Group, German Cancer Research Center (DKFZ), D-69120 Heidelberg, Germany

[13] Nikon Imaging Center at the University of Heidelberg, Bioquant, D-69120 Heidelberg, Germany

[14] Division of Molecular Thoracic Oncology, German Cancer Research Center (DKFZ), D-69120 Heidelberg, Germany

[15] These authors contributed equally.

[16] These authors jointly supervised this work.

* Corresponding author: sergio.acebron@cos.uni-heidelberg.de

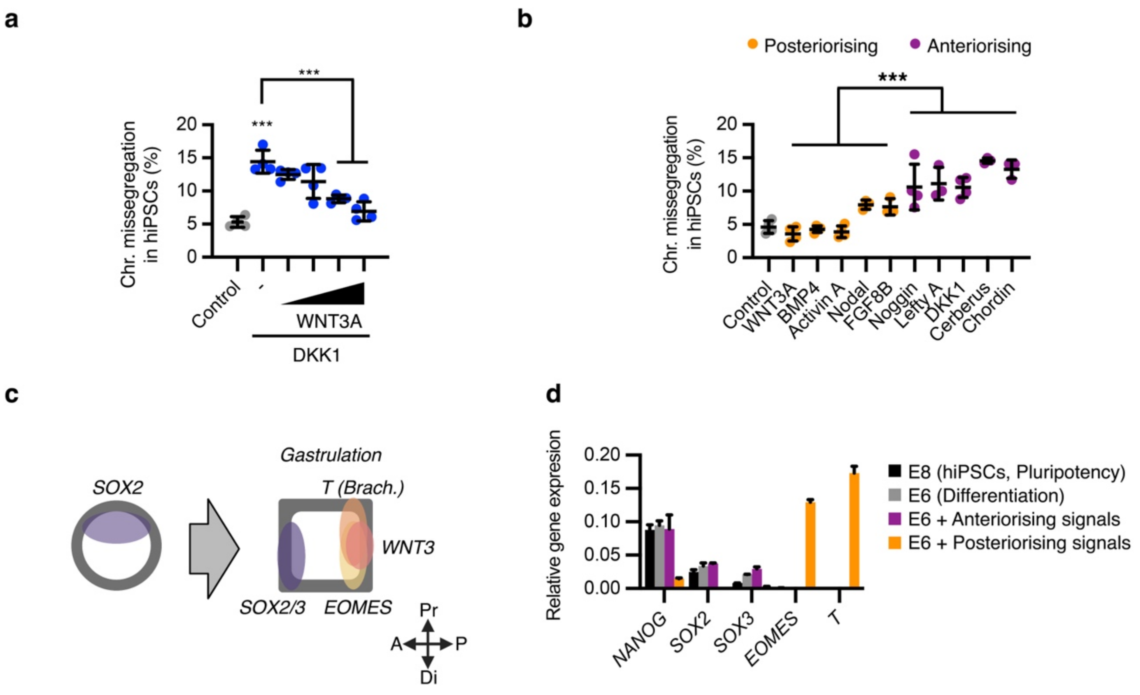

Supplementary Fig. 1: Validation of WNT-DKK1 gradients and signals driving anteriorisation and posteriorisation using hiPSC culture

a Chromosome segregation analyses in hiPSCs upon co-treatment for 16 hours with DKK1 and different dilutions of WNT3A conditioned medium (1:50, 1:25, 1:10, 1:4). Data are mean ± s.d. of n = 4 biological replicates, with > than 100 anaphases analysed in each condition per replicate). *P*-value from one-way ANOVA analyses with multiple comparisons with Dunnet corrections are indicated as ***P < 0.001. **b** Detail of chromosome segregation analyses in hiPSCs from Fig. 1b upon individual treatment with the indicated signals associated with posteriorisation or anteriorisation during mammalian gastrulation (Data extracted from Fig. 1b). Data are mean ± s.d. of n > 3 biological replicates, with > than 100 anaphases analysed in each condition per replicate). *P*-value from a two-sided t-test between the two groups is indicated as ***P = 0.000008. **c** Model for the expression patterns of SOX2/3, EOMES, WNT3 and Brachyury (T) in mammals during gastrulation. **d** Representative experiment of a qRT-PCR of hiPSCs cultured in E8 media (pluripotency) or treated for 16 hours with E6 media (differentiation); E6 media supplemented with the anteriorising signals Noggin, LEFTY2, DKK1, Chordin and Cerberus; or E6 media supplemented with the posteriorising signals WNT3A, BMP4, Nodal and FGF8B. Data are mean ± s.d. of n = 3 technical replicates. This experiment was reproduced twice. Source data for all experiments are provided as a Source data file. Supplementary Fig. 1c was created with BioRender.com released under a Creative Commons Attribution-NonCommercial-NoDerivs 4.0 International license [1].

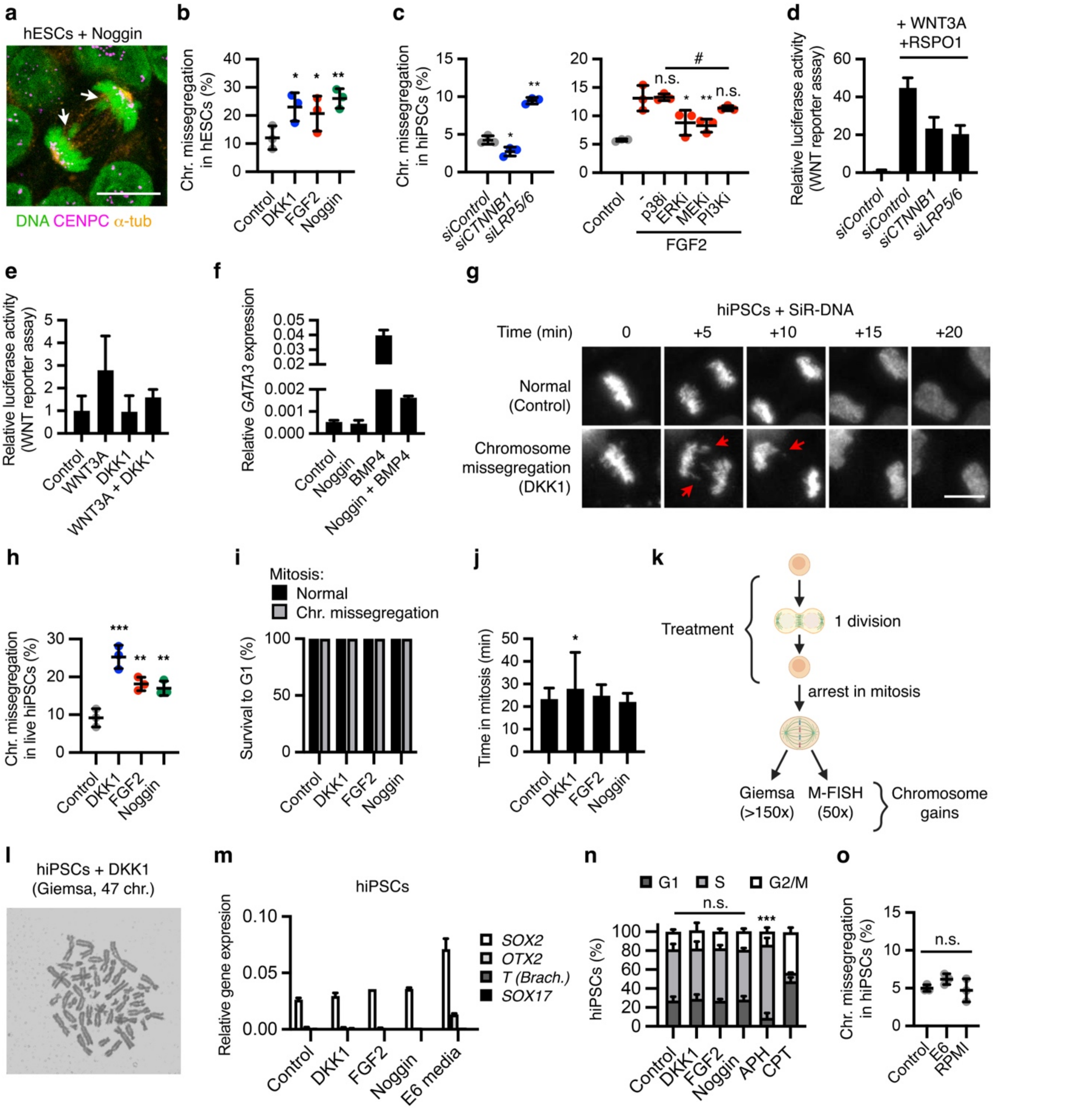

Supplementary Fig. 2: WNT, BMP and FGF regulate chromosome segregation in hESCs and hiPSCs

a,b Chromosome segregation analyses in human embryonic stem cells (hESCs) upon treatment with the indicated signals for 16 hours. Data are mean ± s.d. of n = 3 biological replicates, with > than 100 anaphases analysed in each condition per replicate. *P*-values from one-way ANOVA analyses with multiple comparisons with Holm-Šidák corrections are indicated from left to right as *P = 0.017, *P = 0.027, and **P = 0.0065. **c** Chromosome segregation analyses in hiPSCs upon knockdown or treatment with small compounds targeting downstream components of WNT and FGF signalling respectively, as indicated. Data are mean ± s.d. of n = 3 biological replicates, with > than 100 anaphases analysed in each condition per replicate. *P*-values from one-way ANOVA analyses with multiple comparisons with Tukey corrections are indicated are indicated as *P < 0.05, **P < 0.01, or n.s. (P > 0.05, not significant). Samples under # were compared with FGF2 treatment. **d,e** WNT reporter assays upon the knock down or treatment with the indicated factors. Data are mean ± s.d. of n = 3-4 biological replicates. **f,** Representative qRT-PCR analyses of hiPSCs treated for 6 hours as indicated. Data are mean ± s.d. of n = 3 technical replicates. This experiment was replicated three times. **g-j** Live cell imaging analyses of mitotic hiPSCs labelled with SiR-DNA and treated as indicated. In **(g)**, Data are mean

± s.d. of n = 3 biological replicates. In **(i, j)**, time of mitosis was calculated from chromosome condensation to the end of anaphase.In **(i)** data corresponds to mitosis analysed from the three biological replicates from **(h)**; Control (n= 82 cells), DKK1 (n= 24 cells), FGF2 (n= 62 cells), Noggin (n= 61 cells). *P*-values from one-way ANOVA analyses with multiple comparisons with Tukey corrections are indicated are indicated from left to right as ***P < 0.0001, **P = 0.0042, and **P = 0.0091. **k** Experimental setup for the karyotype analyses shown in Fig. 2g. **l** Example of Giemsa staining of a hiPS aneuploid cell treated with DKK1. **m** Representative qRT-PCR analyses of hiPSCs treated for 16 hours as indicated. Data are mean ± s.d. of n = 3 technical replicates. This experiment was repeated twice. **n** Cell cycle flow cytometry analyses of hiPSCs treated as indicated. APH, aphidicolin; CPT, Camptothecin. Data are mean ± s.d. of n = 3-6 biological replicates except for CPT condition (n= 2 biological replicates). *P*-values from one-way ANOVA analyses with multiple comparisons with Tukey corrections are indicated are indicated as ***P < 0.0001, or n.s. (P > 0.05, not significant). **o** Chromosome segregation analyses in hiPSCs upon culture for 16 hours in control medium (E8) or differentiating media (E6, RPMI). Data are mean ± s.d. of n = 3 biological replicates, with > than 100 anaphases analysed in each condition per replicate. *P*-values were calculated from one-way ANOVA analyses with Tukey correction of mean ± s.d. of 3 biological replicates, and indicated as n.s. (P > 0.05, not significant). Scale bars = 10 μm. Source data for all experiments are provided as a Source data file. Supplementary Fig. 2k was created with BioRender.com released under a Creative Commons Attribution-NonCommercial-NoDerivs 4.0 International license [1].

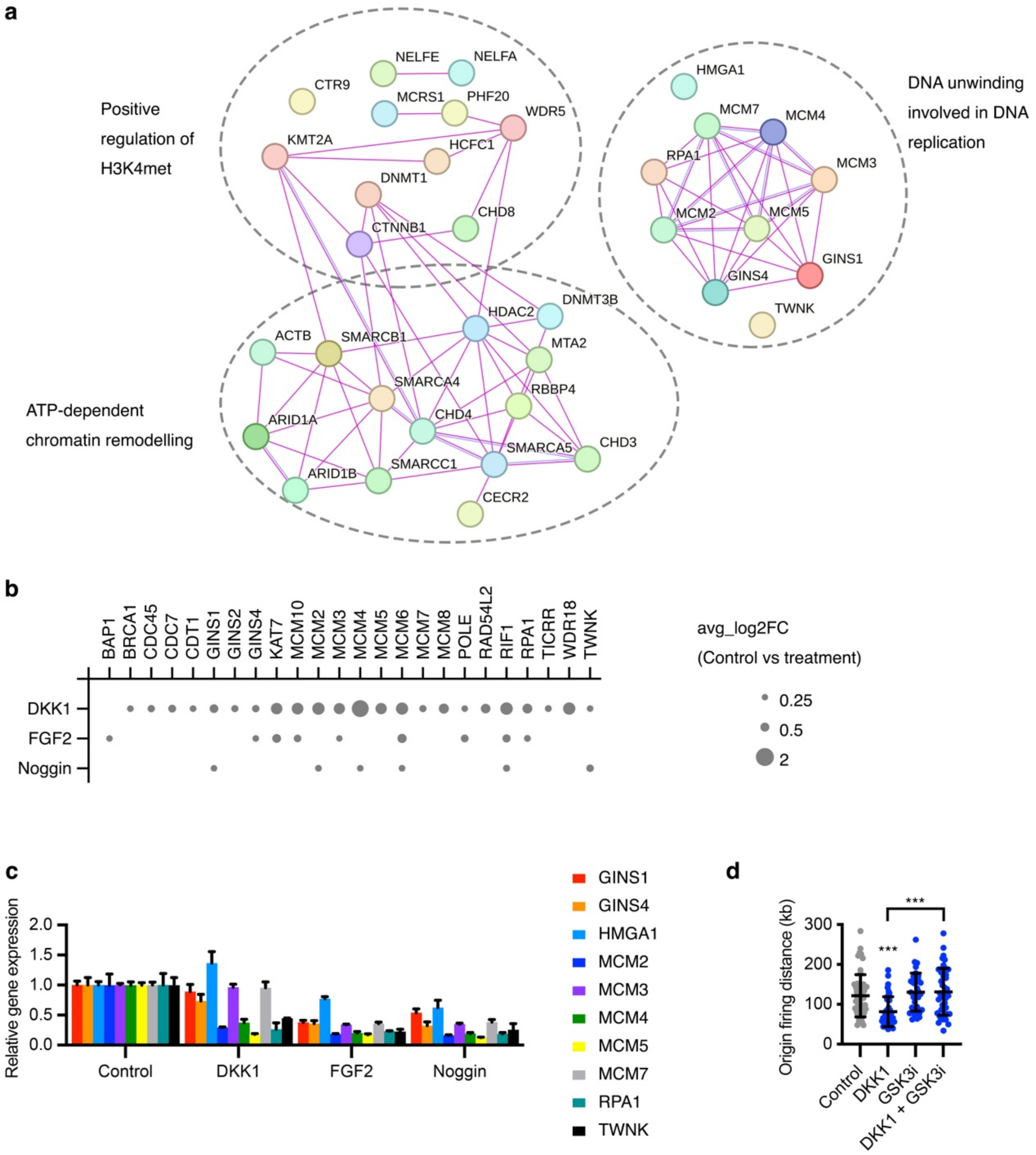

Supplementary Fig. 3: WNT, BMP and FGF converge into the regulation of DNA replication

a STRING network analyses of the factors differentially regulated by WNT, FGF and BMP in the three most prominent GO terms enriched in the single cell sequencing analyses from Fig. 3a, b and Supplementary Data 2. **b** DNA replication factors which expression was differentially regulated by either DKK1, FGF2 or Noggin. Data extracted from Supplementary Data 2 and it is represented as average of the log2FC of every treatment respect Control condition, after sequencing and analysis of n= 74 single cells per condition. **c** Representative qRT-PCR validation analyses of the factors from the GO term "*DNA unwinding involved in DNA replication*" differentially regulated in the single cell sequencing analyses from Fig. 3a, and depicted in **(a)** Data are mean ± s.d. of n = 3 technical replicates. This experiment was repeated three times. **d** Representative DNA combing analyses of inter-origin distances in hiPSCs upon treatment as indicated for 3 hours. Data are mean ± s.d. of inter-origin distances: Control (n= 53), DKK1 (n= 41), GSK3i (n= 34), DKK1 + GSK3i (n= 44). *P*-values from one-way ANOVA analyses with multiple comparisons with Tukey corrections are indicated as ***P < 0.0001. Data for all experiments are provided as a Source data file.

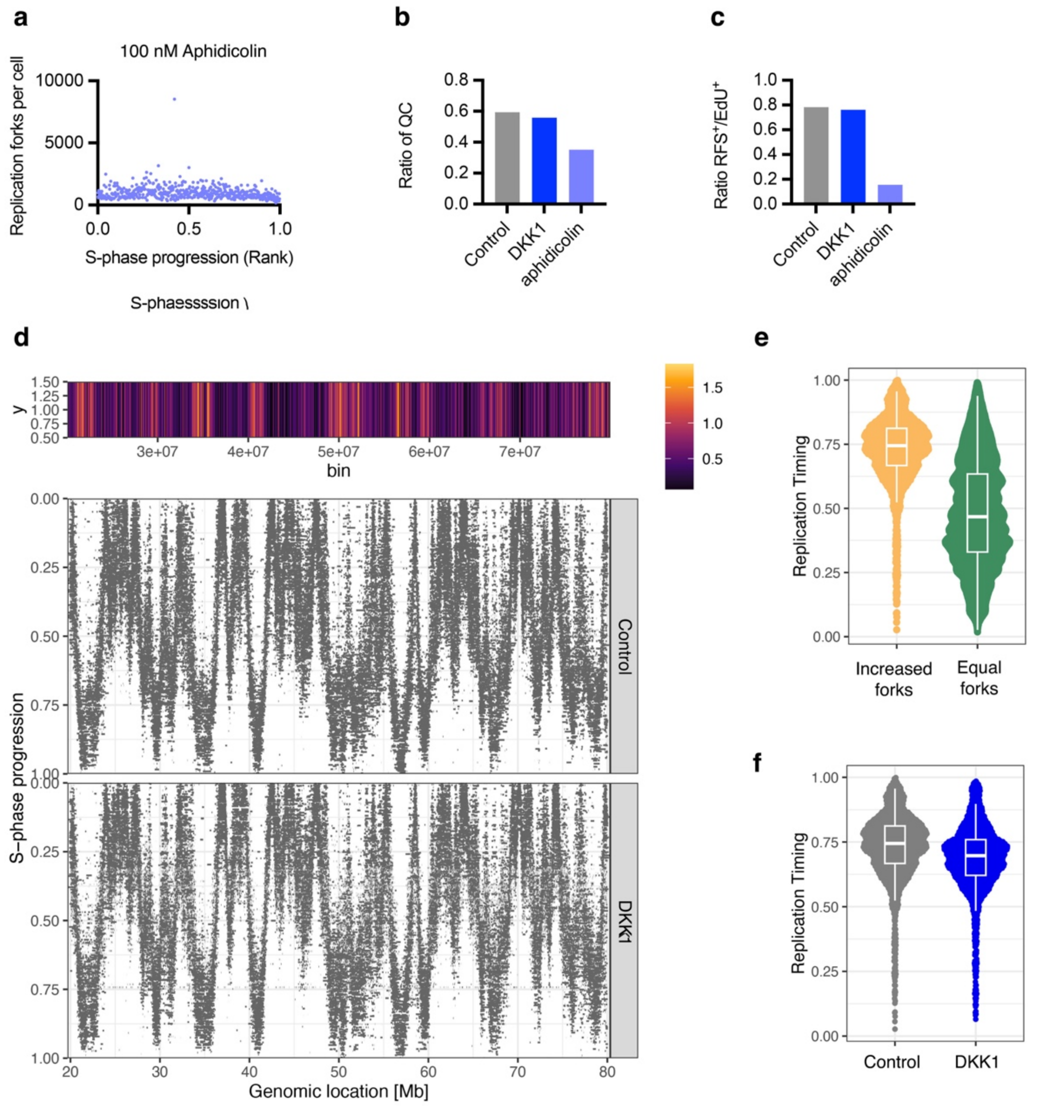

Supplementary Fig. 4: Single cell EdU sequencing analyses in hiPSCs

a Replication forks per cell obtained by scEdU-seq in hiPSCs treated with 100 nM Aphidicolin for 3 hours. N= 529 single cells were ranked for their relative position in S-phase (X-axis) according to their fork distribution pattern across different chromosomes, as previously described [2]. **b,c** Ratios of QC and RFS/EdU positive cells in hiPSCs treated as indicated. **d** Top – Difference in median replication timing (log_2(DKK1/Control) per 100 kb bin for the indicated region of chromosome 2. Bottom – DNA replication tracks for both Control and DKK1-treated hiPSC cells for the indicated representative region of chromosome 2. Note that excess of forks (upper panel, orange) coincides with areas of fork conversion (lower panel). **e** Quantification of the replication timing of bins with equal forks or increased forks (log_2fold change >1) in DKK1 compared to Controls. Note that cells showing increased number of forks are enriched at mid/late S-phase (Rank ~0.75). **f** Replication Timing of bins which display increased number of forks in DKK1 treated cells compared to control hiPSCs. Note that the data suggests a mild decrease in replication timing of bins with increased replication forks in DKK1 treated cells. Source data for all experiments are provided as a Source data file.

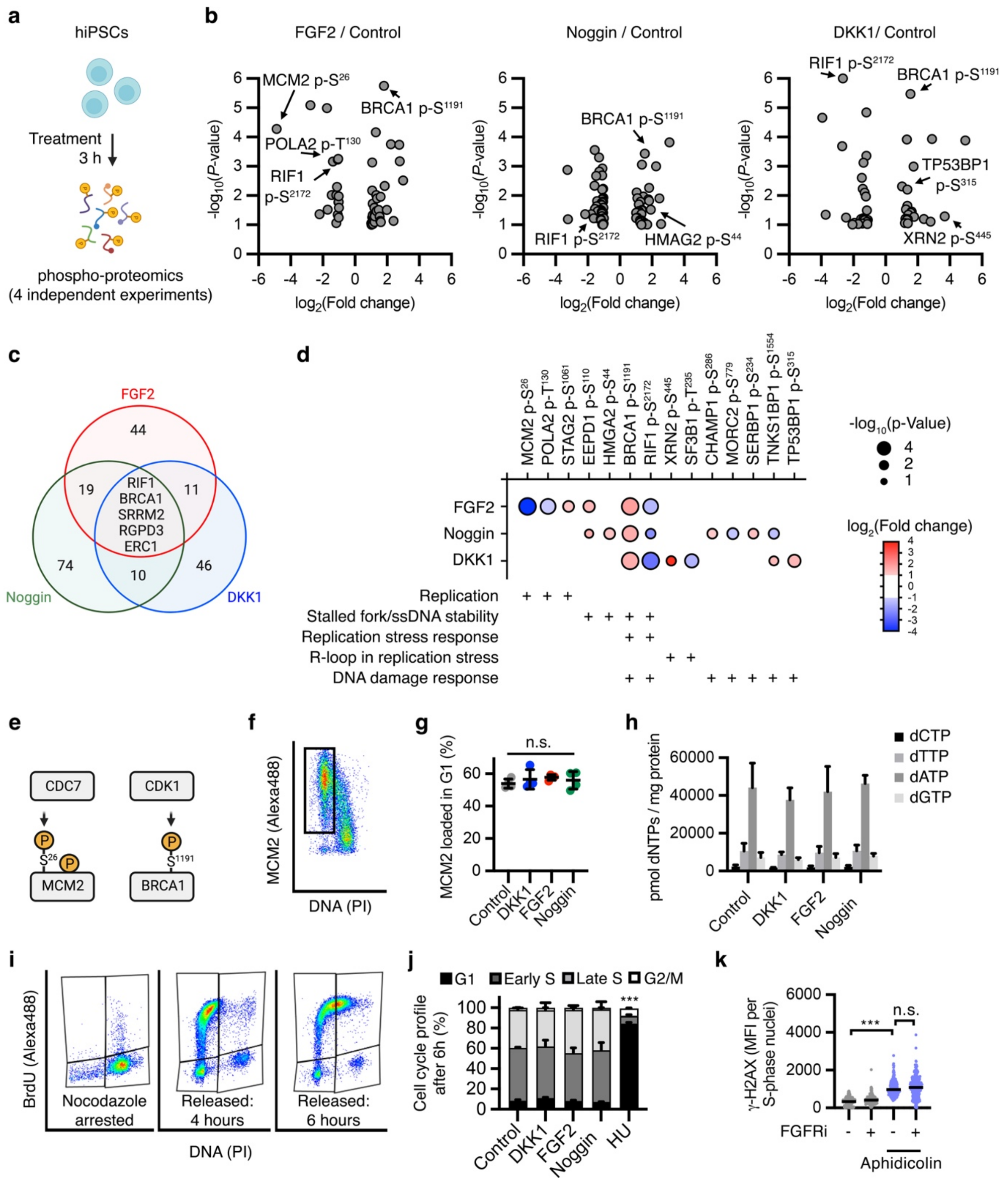

Supplementary Fig. 5: Proteomics and epistasis analyses of WNT, BMP and FGF in DNA replication

a Schematic of the phospho-proteomics analysis. **b** Differentially regulated phospho-peptides by DKK1, Noggin and FGF2 in hiPSCs after 3 hours (n= 4 biological replicates per condition). Note that DNA replication and damage response proteins are among most significantly regulated factors by either treatment. **c** Venn diagram of differentially regulated proteins in the phospho-proteome analysis. **d** Differentially regulated factors from the phospho-proteomics experiment that are associated with DNA replication and/or damage response. The list was hand-curated by searching each differentially regulated proteins for known roles in those processes (See Supplementary Data 3). Data in **(b,d)** is represented as log_2FC respect Control condition, including the $-log_{10}$ P-value (Further detailed in methods). **e** Schematic with two known kinase-target associations. **f,g** FACS analyses of total MCM2 loading (protein) levels across the cell cycle in unarrested hiPSCs treated as indicated for 3 hours. Data are mean ± s.d. of n = 3 biological replicates, with > than 20.000 cells analysed in each condition per replicate. *P*-values from one-way ANOVA analyses with multiple comparisons with Tukey corrections are indicated as n.s. (P > 0.05, not significant). **h** HPLC analyses of dNTP

pools in hiPSCs treated for 3 hours as indicated. Data are mean ± s.d. of n = 3-4 biological replicates. **I,j** FACS analyses of S-phase progression of hiPSCs arrested in mitosis with nocodazole and released and treated for 6 hours as indicated in **(j)** Data in **(j)** are mean ± s.d. of n = 3 biological replicates, with > than 20.000 cells analysed in each condition per replicate). *P*-values from one-way ANOVA analyses with multiple comparisons with Tukey corrections are indicated as ***P < 0.001. **k** Accumulation of γ-H2AX foci in hiPSCs treated for 3 hours as indicated. Data are median fluorescence intensity (MFI) of Control (n= 263), Aphidicolin (n= 253), Aphidicolin + FGFRi (n= 303), FGFRi (n= 213) EdU^+ nuclei from a representative experiment. *P*-values from one-way ANOVA analyses with multiple comparisons with Tukey corrections are indicated as ***P < 0.0001 and n.s. (P = 0.14). Source data for all experiments are provided as a Source data file. Supplementary Fig. 5a, e were created with BioRender.com released under a Creative Commons Attribution-NonCommercial-NoDerivs 4.0 International license [1].

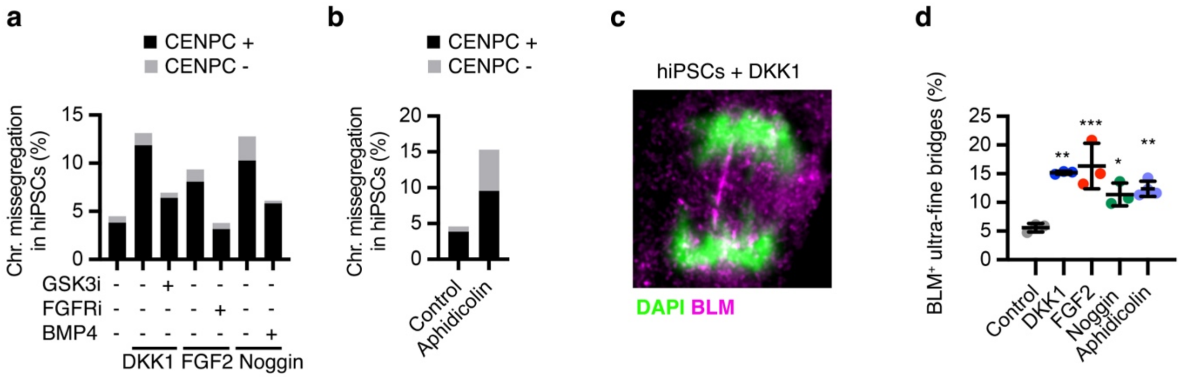

Supplementary Fig. 6: WNT and BMP inhibition, as well as FGF activation, result in structural chromosomal defects during anaphase

a Analysis of the missegregated chromosomes with or without CENPC labelling from 4 independent experiments (N > 380 cells per condition) shown in Fig. 2b, and treated as indicated for 16 hours. Control (n= 753), DKK1 (n= 808), DKK1+GSK3i (n= 385), FGF2 (n=645), FGF2+FGFRi (n= 493), Noggin (n= 544), Noggin + BMP4 (n= 382). **b** Analysis of the CENPC positive and negative missegregated chromosomes in presence or absence of 50 nM aphidicolin for 16 hours. **c,d** Quantification of ultrafine bridges (UFBs) in human induced pluripotent stem cells upon treatment with the indicated signals, or 50 nM aphidicolin for 16 hours. Data are mean ± s.d. of n = 3-4 biological replicates, with > than 50 anaphases analysed in each condition per replicate). *P*-values from one-way ANOVA analyses with multiple comparisons with Tukey corrections are indicated as *P < 0.05, **P < 0.01, ***P < 0.001. Source data for all experiments are provided as a Source data file.

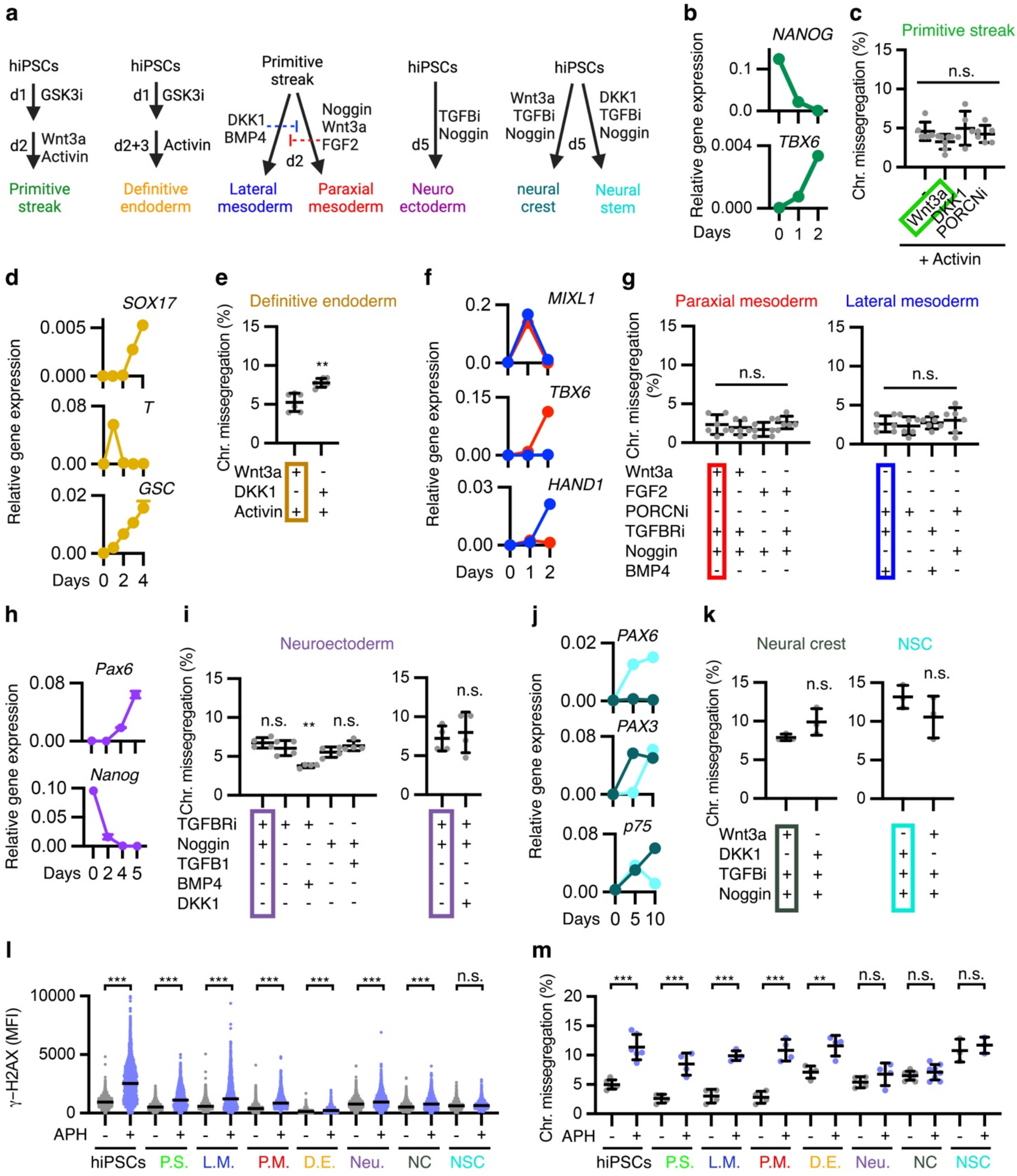

Supplementary Fig. 7: WNT, BMP and FGF display limited functions in chromosome segregation fidelity in early human specified lineages

a Summary of the treatments and days (d) required to generate different lineages from hiPSCs. **b-k** Cell lineage specification experiments performed as indicated in **(a)**. In **(b, d, f, h, j)** cells were harvested at the indicated differentiation days and analysed by qRT-PCR for lineage-specific markers**.** Representative experiments are shown as mean ± s.d. of n = 3 technical replicates (each experiment was reproduced > 2 times). In **(c, e, g, i, k)** differentiating media was modified as indicated during the last 16 h and chromosome segregation was analysed by immunofluorescence. Data are mean ± s.d. of n= 4-6 biological replicates with >100 anaphases analysed per condition and per replicate. *P*-values from one-way ANOVA analyses with multiple comparisons with Tukey corrections are indicated as *P < 0.05, **P < 0.01, ***P < 0.001, or n.s. (P > 0.05, not significant). **l, m** Impact of aphidicolin (APH)-induced DNA replication stress in DNA damage and

chromosome missegregation in the indicated lineages, generated as shown in (**a**). In **(l)**, data are median fluorescence intensity (MFI) of γ-H2AX of n=300-3500 nuclei of > than two biological replicates. In **(m)**, data are mean ± s.d. of n= 3-9 biological replicates with > 100 anaphases analysed per condition and per replicate. *P*-values from one-way ANOVA analyses with multiple comparisons with Tukey corrections are indicated as **P < 0.01, ***P < 0.001, or n.s. (P > 0.05, not significant). Primitive streak-like (P.S.), lateral mesoderm (L.M.) paraxial mesoderm (P.M.), definitive endoderm (D.E.), neuroectoderm (Neu), neural crest (NC) and neural stem cells (NSC) are shown. Colour boxes indicate the standard conditions for differentiation into the indicated lineages. Source data for all experiments are provided as a Source data file. Supplementary Fig. 7a was created with BioRender.com released under a Creative Commons Attribution-NonCommercial-NoDerivs 4.0 International license [1].

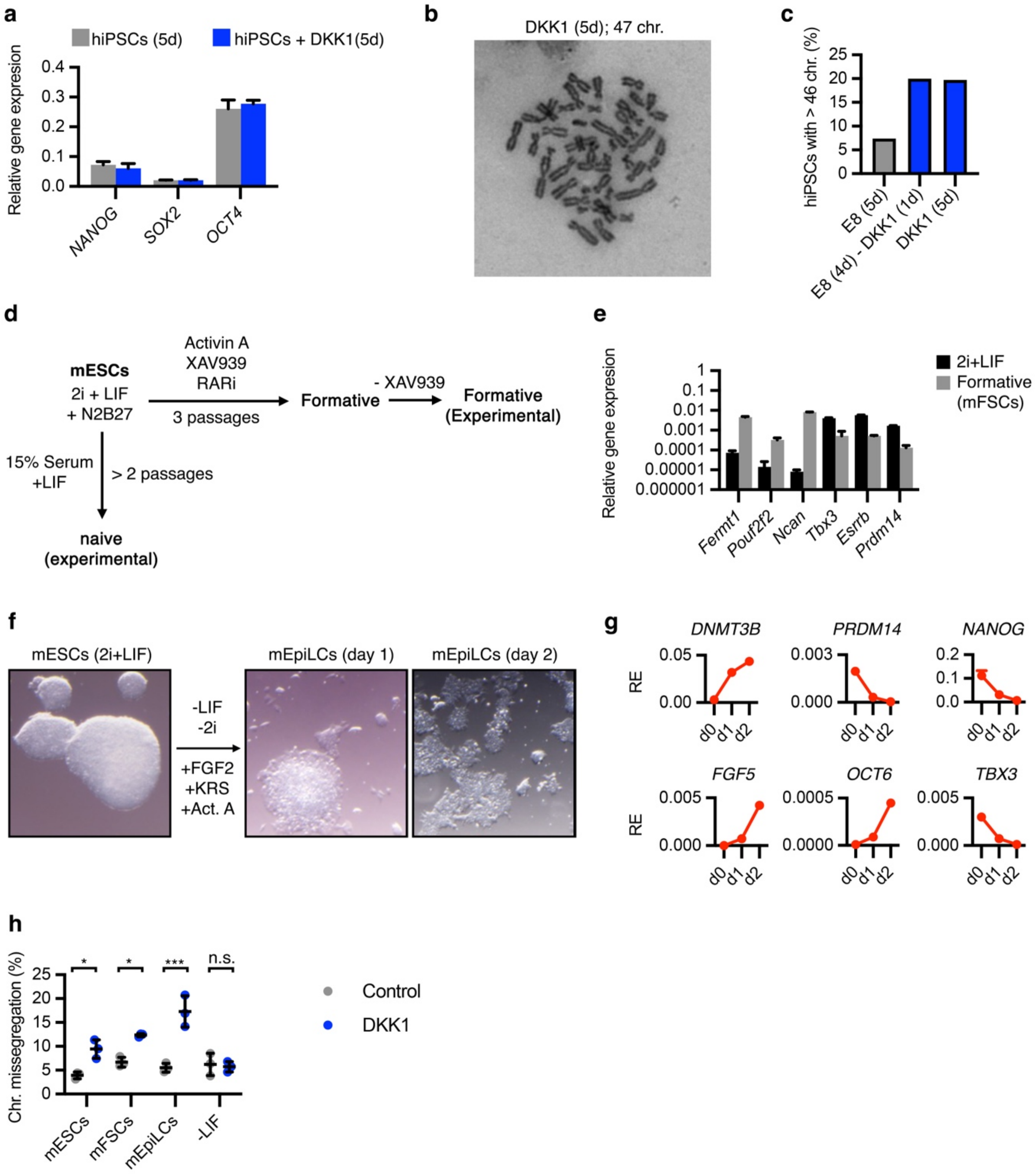

Supplementary Fig. 8: WNT signalling regulates chromosome segregation fidelity in primed hiPSCs, as well as in naive, primed and formative mouse stem cells.

a Representative qRT-PCR analyses of hiPSCs cultured for 5 days in E8 media (pluripotency) or E8 media with DKK1. Data are shown as mean ± s.d. of n = 3 technical replicates. **b,c** Representative karyotype analyses of hiPSCs cultured for 5 days in E8 media, or 1-5 days in E8 media with DKK1; Control (n= 54 metaphases), DKK1-1day (n= 45 metaphases), DKK1-5 days (n= 76 metaphases). **d** Scheme showing the culture conditions of mESCs (naive) for the chromosome segregation analyses, as well as their transition towards mFSCs (Formative). Please note that in the Experimental conditions, we remove any molecule targeting WNT after specification to specifically study the effects of WNT inhibition by DKK1. **e** Representative qRT-PCR analyses of mESCs and mFSCs. Note that the y-axis is shown as $\log_{10}$ scale. Data are shown as mean ± s.d. of n = 3 technical replicates. **f,g** mESCs conversion into primed mouse epiblast-like cells (mEpiLCs) for 2 days; representative qRT-PCR analyses of mESCs to mEpiLCs transition; data of one biological replicate are shown as mean of n = 3 technical replicates, after being successfully reproduced in n= 3 biological independent experiments. **h** Chromosome segregation analyses of naive (mESCs), primed (mEpiLCs), formative (mFSCs)

and differentiated (-LIF) stem cells treated as indicated during the last 16 hours. Data are mean ± s.d. of n= 3 biological replicates with >100 anaphases analysed per condition and per replicate. *P*-values from one-way ANOVA analyses with multiple comparisons with Tukey corrections are indicated from left to right as *P = 0.021, *P < 0.017, ***P < 0.0001, and n.s. (P > 0.99, not significant). Source data for all experiments are provided as a Source data file.

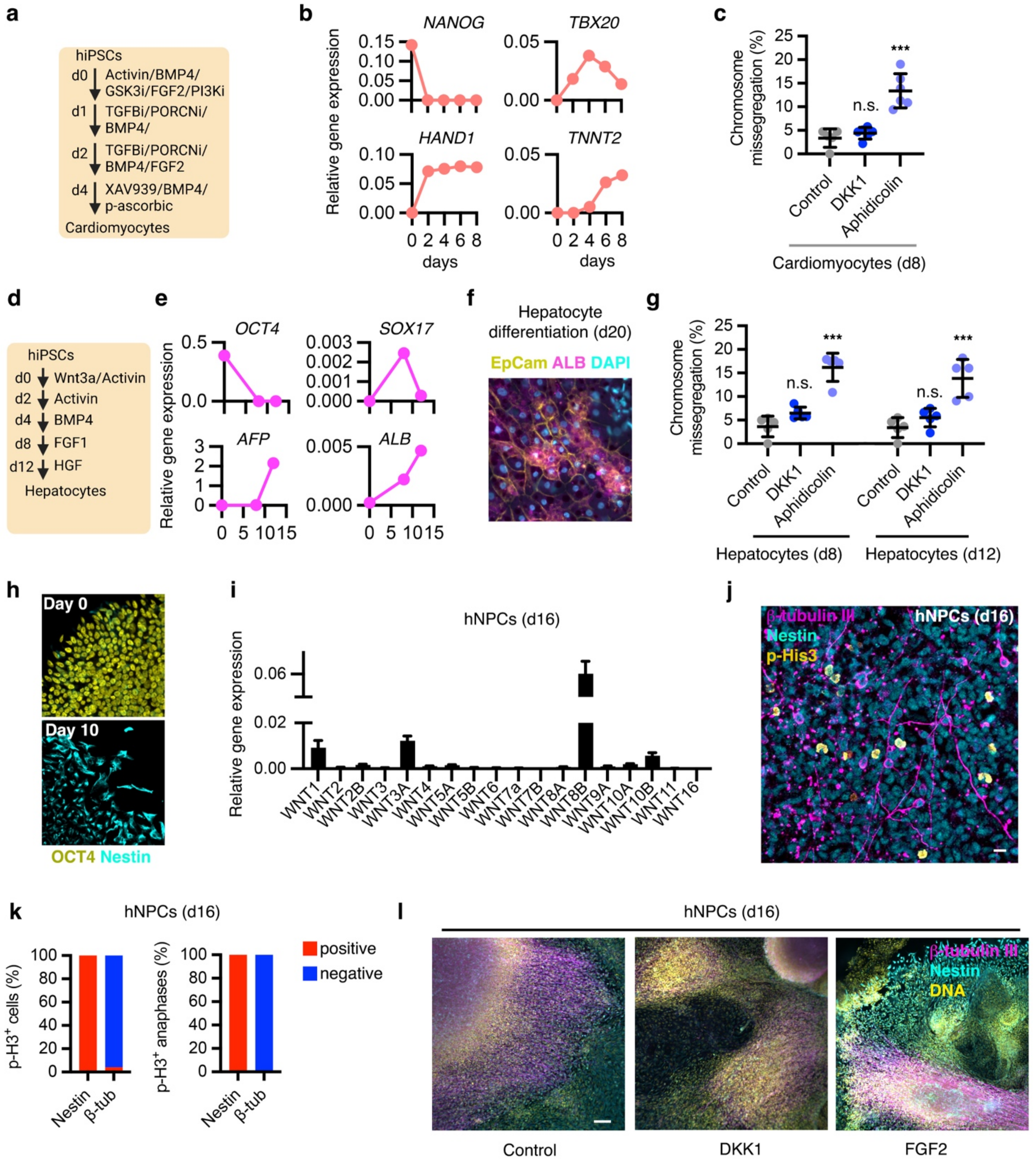

Supplementary Fig. 9: *In vitro* differentiation of hiPSCs into hepatocyte-, cardiomyocyte-like cells and neural progenitors (hiNPCs)

a Summary of the treatments and days required to differentiate hiPSCs into cardiomyocyte-like cells. **b** Representative qRT-PCR analyses of hiPSCs undergoing differentiation into cardiomyocytes. Data are shown as mean ± s.d. of n = 3 technical replicates. **c** Chromosome segregation analyses of *in vitro* generated cardiomyocyte-like cells treated for 16 hours as indicated. Data are mean ± s.d. of n= 6 biological replicates with >100 anaphases analysed per condition and per replicate. *P*-values from one-way ANOVA analyses with multiple comparisons with Tukey corrections are indicated as ***P < 0.0001, or n.s. (P > 0.05, not significant). **d** Summary of the treatments and days required to differentiate hiPSCs into hepatocyte-like cells. **e** Representative qRT-PCR analyses of hiPSCs undergoing differentiation into hepatocytes. Data are shown as mean ± s.d. of n = 3 technical replicates. **f** Representative image of the immunofluorescence analysis of immature hepatocyte-like cells at day 20 of differentiation. **g** Chromosome segregation analyses of *in vitro*

generated hepatocyte-like cells treated for 16 hours as indicated. Data are mean ± s.d. of n= 5 biological replicates with >100 anaphases analysed per condition and per replicate. *P*-values from one-way ANOVA analyses with multiple comparisons with Tukey corrections are indicated as ***P < 0.0001, or n.s. (P > 0.05, not significant). **h** Representative image of the immunofluorescence analyses of hiPSCs (Day 0) and expanding hiNPCs (Day 10). **i** Representative qRT-PCR analyses of WNT ligands in hiNPCs at day 16 of differentiation. Data are shown as mean ± s.d. of n = 3 technical replicates. **j-l** Immunofluorescence analyses of Nestin and beta-tubulin III of the mitotic hNPCs (phospho-Ser10 Histone 3; pHis3) used in the chromosome segregation analyses. n= 247 cells (from three biological replicates). In (**l**) hiNPCs at day 16 of differentiation were treated for 16 hours as indicated. Scale bars = 10 µm **(j)** and 100 µm **(l).** Source data for all experiments are provided as a Source data file.

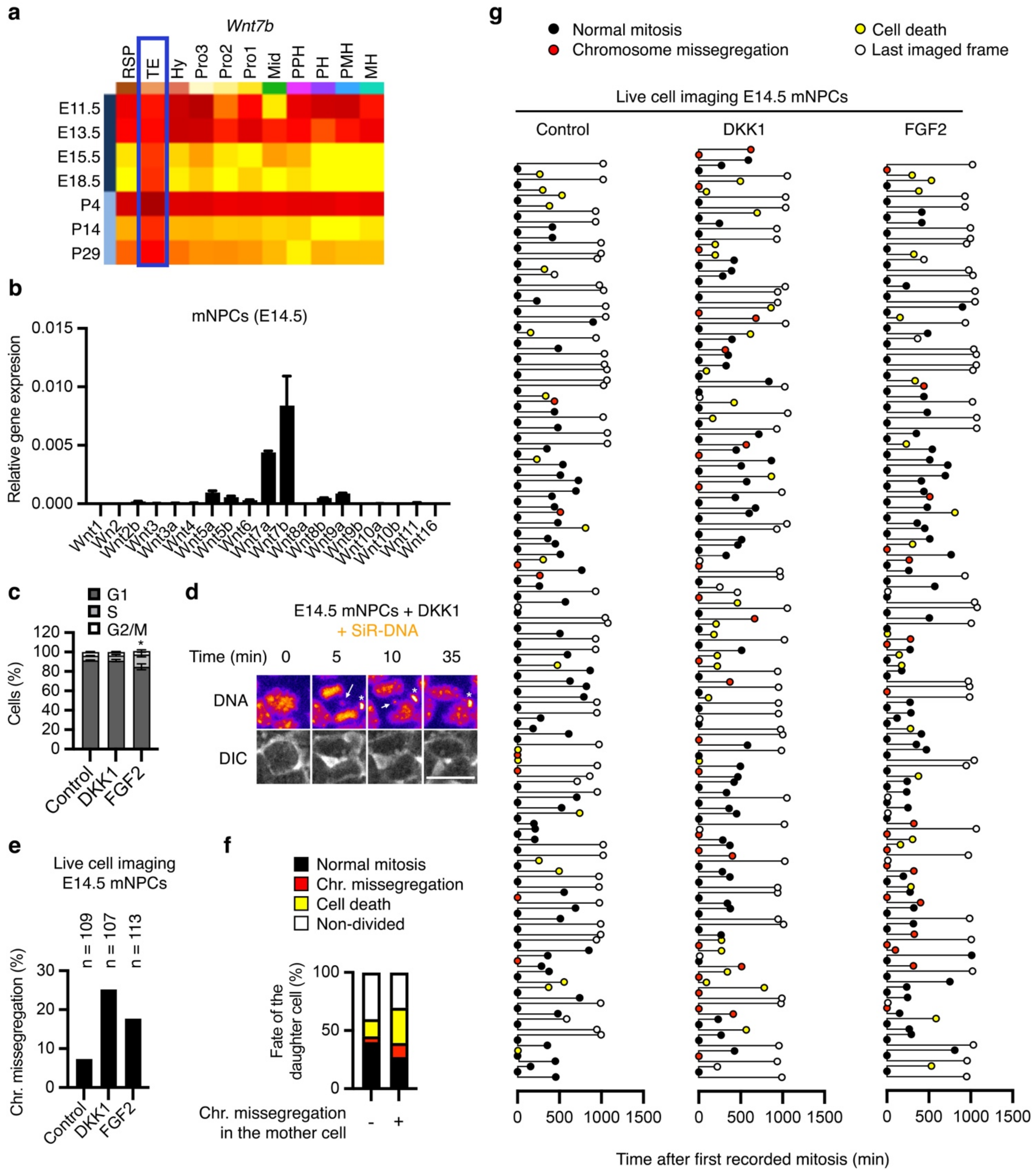

Supplementary Fig. 10: Signalling and chromosome segregation analyses in mouse NPCs

a Expression profile of *Wnt7b* in the developing nervous system obtained from the Allen Developing Mouse Brain Atlas (http://developingmouse.brain-map.org/). RSP: rostral secondary prosencephalon; TE: telencephalon (highlighted); Hy: peduncular (caudal) hypothalamus; Pro3: prosomere 3; Pro2: prosomere 2; Pro1: prosomere 1; Mid: midbrain; PPH: prepontine hindbrain; PH: pontine hindbrain; PMH: pontomedullary hindbrain; MH: medullary hindbrain (medulla). **b** Representative qRT-PCR analyses of WNT ligands in NPCs isolated from E14.5 mouse embryos. Data are shown as mean ± s.d. of n = 3 technical replicates. Experiment was replicated twice with similar results. **c** Cell cycle profiles of mNPCs isolated from E14.5 embryos and treated for 16h as indicated. Note that, as previously reported, FGF2 promotes proliferation in mNPCs. Data are mean ± s.d. of n= 3 biological replicates with >20000 cells measured per condition and per replicate. *P*-values from one-way ANOVA analyses with Tukey correction are indicated as *P < 0.05. **d-g** Representative

live cell imaging analyses of NPCs isolated from E14.5 mouse embryos and stained with SiR-DNA. In (**d**), an exemplary mitosis of NPCs treated with DKK1 is shown. In **(e)** Control (n= 109 anaphases), DKK1 (n= 107 cells), FGF2 (n= 113 cells); data results from two independent experiments. The arrow indicates a lagging chromosome and the asterisk marks an artefact. In (**f, g**), the fate of daughter cells following a normal or chromosome missegregation division in the mother cell was tracked during the recording (>20 hours). In (**f**), all tracked divisions (n = 350) from control-, DKK1- and FGF2-treated mother cells are shown. Note that chromosome missegregation in mother cells do not change the proportion of daughter cells committed to division (Normal mitosis + Chr. Missegregation). In (**g**), single tracks for the daughter cells of divisions in each individual treatment are shows. Scale bar = 10 µm. Source data for all experiments are provided as a Source data file.

Supplementary figure references

1 https://creativecommons.org/licenses/by-nc-nd/4.0/deed.en. Creative Commons Attribution-NonCommercial-NoDerivs 4.0 International license.

2 van den Berg, J. *et al.* Quantifying DNA replication speeds in single cells by scEdU-seq. *Nat Methods* (2024). https://doi.org:10.1038/s41592-024-02308-4
