# Supplementary material for: Developmental signals control chromosome segregation fidelity during pluripotency and neurogenesis by modulating replicative stress
Source: Nat Commun. 2024 Aug 28;15:7404. doi: 10.1038/s41467-024-51821-9 (PMC11350214; doi:10.1038/s41467-024-51821-9)
Supplement: Supplementary file 3 — Description of additional supplementary files [file 41467_2024_51821_MOESM3_ESM.pdf]

Description of Additional Supplementary Files

Supplementary Data 1 : Technical details of the proteins and small compounds used in the chromosome segregation screen, as well as their potential role in gastrulation.

Supplementary Data 2 : Differentially expressed genes (DEG) and Gene Ontology (GO) analyses in hiPSCs treated with DKK1, FGF2 or Noggin and analysed by single cell sequencing.

Supplementary Data 3 : Phospho-proteomics analyses in hiPSCs treated with DKK1, FGF2 or Noggin for 3 hours.

Supplementary Data 4 : List of human and mouse qPCR primers and list of siRNAs employed.
