# Supplementary material for: Developmental signals control chromosome segregation fidelity during pluripotency and neurogenesis by modulating replicative stress
Source: Nat Commun. 2024 Aug 28;15:7404. doi: 10.1038/s41467-024-51821-9 (PMC11350214; doi:10.1038/s41467-024-51821-9)
Supplement: Supplementary file 8 — Reporting Summary [file 41467_2024_51821_MOESM8_ESM.pdf]

Corresponding author(s): Sergio P. Acebron
Last updated by author(s): Jul 16, 2024

Reporting Summary

Nature Portfolio wishes to improve the reproducibility of the work that we publish. This form provides structure for consistency and transparency in reporting. For further information on Nature Portfolio policies, see our Editorial Policies and the Editorial Policy Checklist.

Statistics

For all statistical analyses, confirm that the following items are present in the figure legend, table legend, main text, or Methods section.

n/a	Confirmed	
☐	☒	The exact sample size (*n*) for each experimental group/condition, given as a discrete number and unit of measurement
☐	☒	A statement on whether measurements were taken from distinct samples or whether the same sample was measured repeatedly
☐	☒	The statistical test(s) used AND whether they are one- or two-sided<br>*Only common tests should be described solely by name; describe more complex techniques in the Methods section.*
☒	☐	A description of all covariates tested
☐	☒	A description of any assumptions or corrections, such as tests of normality and adjustment for multiple comparisons
☐	☒	A full description of the statistical parameters including central tendency (e.g. means) or other basic estimates (e.g. regression coefficient) AND variation (e.g. standard deviation) or associated estimates of uncertainty (e.g. confidence intervals)
☐	☒	For null hypothesis testing, the test statistic (e.g. F, t, r) with confidence intervals, effect sizes, degrees of freedom and P value noted<br>*Give P values as exact values whenever suitable.*
☒	☐	For Bayesian analysis, information on the choice of priors and Markov chain Monte Carlo settings
☒	☐	For hierarchical and complex designs, identification of the appropriate level for tests and full reporting of outcomes
☒	☐	Estimates of effect sizes (e.g. Cohen's d, Pearson's r), indicating how they were calculated

Our web collection on statistics for biologists contains articles on many of the points above.

Software and code

Policy information about availability of computer code

Data collection

Images were collected at Nikon Imaging Center (Heidelberg) and COS Heidelberg, using the following microscopes: NiE epifluorescence microscope, Nikon AX Confocal microscope, SP8 confocal microscope (Leica Microsystems), and Nikon CREST microscope (for live cell imaging experiments).

Single cell RNA sequencing datasets were processed and analyzed using the following tools:
Read alignments and the count tables of mapped read per gene were obtained using STAR version 2.6.0a
For differential gene expression analysis, the R package Seurat v 4.1.1 133 was applied.
After normalizing the data for differences in library size, the 'FindMarkers' function with the ROC test was used to determine differentially expressed genes (DEGs) between the different treatment conditions.
We used DAVID for Gene Ontology analysis.

Phospho proteomics datasets were processed and analyzed using the following approach:
Data analysis was carried out by MaxQuant (version 1.6.14.0). In total 113165 peptides and 8104 proteins could have been identified by MS/MS based on an FDR cutoff of 0.01 on peptide level and 0.01 on protein level. Identified in all samples were 40292 peptides and 6620 proteins. Match between runs option was enabled to transfer peptide identifications across Raw files based on accurate retention time and m/z. Quantification was done using a label free quantification approach based on the MaxLFQ algorithm. A minimum number of quantified peptides was required for protein quantification. In total 7394 proteins could have been quantified, 4140 were quantified in all samples.
For the downstream analysis, only peptides with fold changes > 2 and p-values < 0.1 for the 4 experiments were considered differentially regulated and are shown in Figure S5. Each of the factors were manually analysed in STRING, PhosphoSitePlus, and other databases for i) their role in DNA replication or/and damage, ii) known kinases modulating the identified phospho-sites.

	scEdU-seq was performed as previously described van den Berg et al., Nature Methods 2024. Scripts to process and analyse the data are available at https://github.com/vincentvbatenburg/scEdU-seq.
Data analysis	qPCR data were analyzed by QuantStudio Real Time PCR software. Statistics analysis were calculated by GraphPad Prism v9.0 and v10. Immunofluorescence stainning images and live cell imaging data were processed using ImageJ and NIS Elements.

For manuscripts utilizing custom algorithms or software that are central to the research but not yet described in published literature, software must be made available to editors and reviewers. We strongly encourage code deposition in a community repository (e.g. GitHub). See the Nature Portfolio guidelines for submitting code & software for further information.

Data

Policy information about availability of data

All manuscripts must include a data availability statement. This statement should provide the following information, where applicable:

- Accession codes, unique identifiers, or web links for publicly available datasets
- A description of any restrictions on data availability
- For clinical datasets or third party data, please ensure that the statement adheres to our policy

All data generated in this study are provided in the article file, Supplementary Information, and Supplementary Data. The relevant source data from each figure are provided in the Source Data files.
Single cell RNA-sequencing datasets generated during this study and disclosed in Fig. 3a and Supplementary Fig. 3 are available at ENA database, with accession number: PRJEB76601; https://www.ebi.ac.uk/ena/browser/view/PRJEB76601.
Single cell EdU-sequencing datasets generated during this study and disclosed in Fig. 3j-k and Supplementary Fig. 4 are available at GEO database, with accession number: GSE271478; https://www.ncbi.nlm.nih.gov/geo/query/acc.cgi?acc=GSE271478.
The phospho-MS data generated in this study and represented in Supplementary Fig. 5a-d are provided in the Supplementary Information and in the Source Data file.

Research involving human participants, their data, or biological material

Policy information about studies with human participants or human data. See also policy information about sex, gender (identity/presentation), and sexual orientation and race, ethnicity and racism.

Reporting on sex and gender	Human embryonic stem cell line H9 (female) and human induced pluripotent stem cells (hiPSCs)-male.
Reporting on race, ethnicity, or other socially relevant groupings	*Please specify the socially constructed or socially relevant categorization variable(s) used in your manuscript and explain why they were used. Please note that such variables should not be used as proxies for other socially constructed/relevant variables (for example, race or ethnicity should not be used as a proxy for socioeconomic status).*<br>*Provide clear definitions of the relevant terms used, how they were provided (by the participants/respondents, the researchers, or third parties), and the method(s) used to classify people into the different categories (e.g. self-report, census or administrative data, social media data, etc.)*<br>*Please provide details about how you controlled for confounding variables in your analyses.*
Population characteristics	*Describe the covariate-relevant population characteristics of the human research participants (e.g. age, genotypic information, past and current diagnosis and treatment categories). If you filled out the behavioural & social sciences study design questions and have nothing to add here, write "See above."*
Recruitment	*Describe how participants were recruited. Outline any potential self-selection bias or other biases that may be present and how these are likely to impact results.*
Ethics oversight	Work with human embryonic stem cells (hESCs) was conducted by the group of Marta Shahbazi at the MRC Laboratory of Molecular Biology (LMB) under an approval from the UK Stem Cell Bank Steering Committee, and in accordance with the regulations of the UK Code of Practice for the Use of Human Stem Cell lines. H9 hESCs were kindly provided by M. Lancaster (LMB) under an agreement with WiCell.<br><br>The human induced pluripotent stem cells (hiPSCs) were a gift from Kyung-Min Noh (EMBL).

Note that full information on the approval of the study protocol must also be provided in the manuscript.

Field-specific reporting

Please select the one below that is the best fit for your research. If you are not sure, read the appropriate sections before making your selection.

☒ Life sciences ☐ Behavioural & social sciences ☐ Ecological, evolutionary & environmental sciences

For a reference copy of the document with all sections, see nature.com/documents/nr-reporting-summary-flat.pdf

Life sciences study design

All studies must disclose on these points even when the disclosure is negative.

Sample size	Initial pilot experiments were carried out to estimate the intravariability of the experimental conditions and to determine the optimal sample

Sample size	size for each experiment, suitable for detecting statistically significant differences. The exact sample size of each experiment is indicated in the figure legend of every Figure.
Data exclusions	No data was excluded
Replication	Data is represented in all the experiments by at least n= 3 biological experimental replicates after successful independent confirmation of the results. In some figures, representative experiments are shown (as indicated in the figure legend). Live cell maging ex vivo experiments using mouse NPCs were replicated twice. In utero intra-ventricular injection was performed in 3 embryos per condition, and 10 sections of each were analyzed.
Randomization	Prior in utero intra-ventricular injection was performed, embryos were randomize in two groups (for injection of PBS or DKK1). For the rest of the experiments, randomization did not apply.
Blinding	All the figures including chromosome segregation analysis have been collected by 4 different scientists resulting in consistent results, and in the case of hESCs, performed blindly by other research group. Unbiased analysis of data was carried out wherever possible.

Reporting for specific materials, systems and methods

We require information from authors about some types of materials, experimental systems and methods used in many studies. Here, indicate whether each material, system or method listed is relevant to your study. If you are not sure if a list item applies to your research, read the appropriate section before selecting a response.

Materials & experimental systems

n/a	Involved in the study	
☐	☒	Antibodies
☐	☒	Eukaryotic cell lines
☒	☐	Palaeontology and archaeology
☐	☒	Animals and other organisms
☒	☐	Clinical data
☒	☐	Dual use research of concern
☒	☐	Plants

Methods

n/a	Involved in the study	
☒	☐	ChIP-seq
☐	☒	Flow cytometry
☒	☐	MRI-based neuroimaging

Antibodies

Antibodies used	Antibodies utilised are described in the Methods section of the article.
Validation	Validation of antibodies was carried out following the manufacture indications and suggestions. Every antibody was tested with positive and negative controls before the experiment was done.

Eukaryotic cell lines

Policy information about cell lines and Sex and Gender in Research

Cell line source(s)	The human induced pluripotent stem cells (hiPSCs) were a gift from Kyung-Min Noh (EMBL). The mouse feeder-free embryonic stem cell line Sox1-GFP was a gift from A. Smith (University of Cambridge), and the line E14Tg2a a gift from C. Niehrs (DKFZ). Work with human embryonic stem cells (hESCs) was conducted at the MRC Laboratory of Molecular Biology (LMB) under an approval from the UK Stem Cell Bank Steering Committee, and in accordance with the regulations of the UK Code of Practice for the Use of Human Stem Cell lines. H9 hESCs were kindly provided by M. Lancaster (LMB) under an agreement with WiCell.
Authentication	Cell lines were authenticated by qRT-PCR and immunostaining.
Mycoplasma contamination	All cell lines were routinely checked for mycoplasma contamination and were tested negative.
Commonly misidentified lines (See ICLAC register)	None

Animals and other research organisms

Policy information about studies involving animals; ARRIVE guidelines recommended for reporting animal research, and Sex and Gender in Research

Laboratory animals	E12.5, E13.5 and E14.5 mouse embryos were obtained from C57BL/6N wild type pregnant females .<br>Further experimental procedures with the embryos and research purpose:<br>E13.5 C57BL/6N wild type embryos were injected in utero either with 5 ng/?L DKK1 or with PBS+0,1% BSA control solution. In detail, pregnant mice were anesthetized with isoflurane, the uterine horns were exposed, and 1 ?l of the solution was injected into the

lateral ventricle of each embryo using glass micropipettes. Animals were sacrificed 16 hours later, and embryonic heads isolated in cold PBS, followed by fixation with 4% PFA for 3 days. Afterward, embryonic heads were cryoprotected in 30% sucrose solution and embedded in Tissue-Tek OCT. Embryonic coronal forebrain sections (18µm) were prepared using a Leica CM1950 cryo-microtome at the DKFZ Light Microscopy Core Facility. Cryosections were subjected to antigen retrieval using 1% sodium citrate, blocked in 0.1% PBST, and incubated overnight at 4°C with 1:250 anti-phospho-FGFR1, anti phospho-LRP6 S1490, anti-Nestin or/and anti-phospho-Ser10-Histone 3 (pHis3).
Mouse neural progenitor cells were dissociated from the neocortex of E14.5 mouse embryo brains using the papain dissociation kit (LK003150, Worthington Biochemical Corporation) following the manufacturer instructions. Prior to seeding, 12-well plates with glass coverslips were coated overnight at 4°C using Poly-D-Ornithine (15 g/mL), and rinse 3 times in PBS prior seeding. Dissociated NPCs were seeded at a 500,000 cells/cm2 density in medium consisting of: Neurobasal media supplemented with B27 (1x), Glutamax (1X) and Penicillin/Streptomycin. Media was changed daily and primary cultured cells were harvested after 48h for experiments.

Wild animals: This study did not involve the use of wild animals.

Reporting on sex: Findings apply to both sexes. Embryos were not sequenced for sex determination and, when applicable, were grouped regardless their sex.

Field-collected samples: This study did not involve the use of field-collected samples.

Ethics oversight: Time-mate pregnant C57BL/6N wild type mice for cortical isolations were purchased from Janvier. Animals had ad libitum access to food and water and were kept under a 12 h light - 12 h dark cycle. All animal experiments were approved by the local governing regional council under A.P. supervision. C57BL/6N wild type mice used for in utero injections were maintained and bred at the DKFZ central mouse facility. In utero injection experiments were approved by the local animal welfare committee Regierungspräsidium Karlsruhe following the guidelines from GV-SOLAS (AZ 35-9185.81/G-94/18 from J.A.). For terminal tissue harvesting procedures, pregnant mice were euthanized using cervical dislocation and embryos were decapitated, following the approved animal facility procedures.

Note that full information on the approval of the study protocol must also be provided in the manuscript.

Plants

Seed stocks: N.A.

Novel plant genotypes: N.A.

Authentication: N.A.

Flow Cytometry

Plots

Confirm that:

☒ The axis labels state the marker and fluorochrome used (e.g. CD4-FITC).

☒ The axis scales are clearly visible. Include numbers along axes only for bottom left plot of group (a 'group' is an analysis of identical markers).

☒ All plots are contour plots with outliers or pseudocolor plots.

☒ A numerical value for number of cells or percentage (with statistics) is provided.

Methodology

Sample preparation: Cells were dissociated into single cells, fixed with EtOH 70% for cell cycle cytometry analysis. Further details on the protocol can be found in the methods section of the article.

Instrument: Cells were analyzed using FACS BD Canto I

Software: Anlayze with FlowJo v.11

Cell population abundance: A minimum of 20,000 cells in the final gate of interest (excluding doublets or debris) were analyzed per sample.

Gating strategy

FSC/SSC initial gating was done to remove debris, doublets or aggregated clumps of cells. Appropiate isotype was used to gate the population of interest, before analyzing the samples stained with BrDU antibody.

☒ Tick this box to confirm that a figure exemplifying the gating strategy is provided in the Supplementary Information.
